# Sex, social rank, and nicotine co-administration shape cocaine- and cocaethylene-induced reinstatement in monkeys

**DOI:** 10.3389/fnbeh.2026.1770940

**Published:** 2026-02-18

**Authors:** Brianna F. Roberts, Mia A. Clark, Michael A. Nader, Mia I. Rough

**Affiliations:** Department of Translational Neuroscience, Wake Forest University School of Medicine, Winston-Salem, NC, United States

**Keywords:** cocaethylene, cocaine, drug-food choice, drug-induced reinstatement, nicotine, nonhuman primates, polysubstance use, relapse

## Abstract

**Introduction:**

Cocaine use disorder (CUD) is highly comorbid with alcohol and nicotine use, yet preclinical research rarely models polysubstance use or incorporates clinically relevant variables such as social and biological factors. This study utilized an animal model of relapse, cocaine-induced reinstatement, under a drug vs. food choice procedure; the effect of co-use of nicotine was also examined. Cocaethylene, the active metabolite formed when alcohol and cocaine are co-used, was also examined with and without nicotine co-use.

**Methods:**

Socially housed male (*N* = 12) and female (*N* = 10) cynomolgus monkeys, all with experience self-administering cocaine or cocaethylene under a concurrent drug vs. food schedule of reinforcement, were studied after drug choice was extinguished by studying saline vs. food choice (< 20% drug choice).

**Results:**

In Experiment 1, both cocaine (0.01–0.3 mg/kg, i.v.) and cocaethylene (0.03–0.3 mg/kg, i.v.) pretreatments reliably increased drug-associated choice; dominant monkeys of both sexes showed greater reinstatement following cocaine and cocaethylene pretreatments when compared to subordinates. Cocaine was also more potent than cocaethylene regardless of sex or social rank. In Experiment 2, nicotine (0.01–0.056 mg/kg) was co-administered with saline, cocaine or cocaethylene. Nicotine alone increased drug-associated choice only in females and selectively increased cocaine-induced drug-associated choice only in females, regardless of social rank. Nicotine did not significantly alter cocaethylene-induced reinstatement, although a trending increase was observed in females.

**Discussion:**

Thus, social rank impacts cocaine- and cocaethylene-induced reinstatement, and the effects of nicotine were influenced by sex. This underscores the value of translational models that move beyond single-drug approaches and suggest that especially in women with CUD, abstaining from nicotine would increase the likelihood of remaining abstinent from cocaine.

## 1 Introduction

Cocaine use disorder (CUD) remains a major public health concern and no FDA-approved pharmacotherapies are currently available. CUD is a highly heterogenous disorder, partially characterized by personal health risks, societal and legal implications, and high relapse rates (Degenhardt et al., 2013; Kampman, 2019; Volkow and Boyle, 2018). Craving is a robust predictor of relapse (Hunt et al., 1971; Paliwal et al., 2008; Sinha, 2011), and both stress and drug-associated cues significantly increase the likelihood of re-initiated cocaine use following abstinence (Preston et al., 2009; Preston et al., 2018; Sinha et al., 1999; Sinha et al., 2006). Neuroimaging studies demonstrate that cocaine cues activate brain regions involved in reward, memory, and stress regulation (Fox et al., 2006; Grant et al., 1996; Kilts et al., 2001; Kilts et al., 2004; Waldrop et al., 2010). Importantly, polysubstance use is the clinical norm: approximately 80% of individuals with CUD also use nicotine (Foltin et al., 2021), and nearly 90% co-use alcohol (van Amsterdam et al., 2024). Epidemiological data indicate that a substantial subset of individuals with CUD also concurrently use alcohol and nicotine, highlighting a clinically significant pattern of polysubstance use that remains largely unmodeled in preclinical research (Allen et al., 2024b; Cougle et al., 2016; Mantsch et al., 2016). This gap underscores the need for models that more accurately reflect the complexity of human substance use.

Cocaine and ethanol co-use result in the formation of cocaethylene, a metabolite with a longer half-life than cocaine and similar potency in inhibiting dopamine reuptake (Bradberry et al., 1993; Landry, 1992). Cocaethylene substituted for cocaine in drug discrimination studies (Katz et al., 1992; Woodward et al., 1991) and maintained responding under various schedules of reinforcement with equal potency to cocaine in rhesus monkeys (Allen et al., 2023; Jatlow et al., 1991). In humans, cocaethylene resulted in cocaine-like subjective effects despite being less potent than cocaine (Hart et al., 2000; Perez-Reyes et al., 1994). Although cocaethylene has been evaluated in self-administration models, its contribution to relapse-related behaviors has not been examined preclinically. Understanding how cocaethylene interacts with other substances such as nicotine may clarify mechanisms that heighten relapse vulnerability in polysubstance users. Nicotine is the most prevalent co-used substance with cocaine, and evidence suggests that it can potentiate cocaine’s reinforcing effects (Allen et al., 2024b; Weinberger et al., 2017). Given the high prevalence of nicotine co-use among individuals with CUD, defining its impact on relapse related behaviors is an important translational goal.

Reinstatement paradigms are widely used to model relapse because they allow systematic evaluation of stimuli that trigger drug seeking, including drug primes, cues, and stressors (Bossert et al., 2013; Nicolas et al., 2022; Shaham et al., 2003). Drug-induced reinstatement can model the extent to which cocaine-like subjective effects promote relapse. In preclinical models of cocaine-induced reinstatement, priming doses of cocaine reliably increase drug-seeking behavior, emphasizing the discriminative stimulus effects of cocaine (McFarland and Kalivas, 2001; Roberts et al., 2025; Schmidt et al., 2005). Both preclinical (Jing et al., 2022) and clinical studies (Childress et al., 1999) demonstrate that drug-associated cues reinstate cocaine seeking via mesocorticolimbic circuitry and activate limbic regions linked to craving and reward. Although reinstatement models provide valuable and translational tools for understanding CUD, important gaps remain in their ability to capture the complexity needed for improved clinical relevance.

Historically, reinstatement models have lacked social and biological complexity, yet emerging evidence shows that social and environmental factors can significantly alter reinstatement responding and relapse vulnerability (Rough et al., 2025b). Sex differences are well documented in both preclinical and clinical studies across multiple stages of the substance use cycle, including relapse (Dos Anjos Rosário et al., 2022; Morales-Silva et al., 2024; Nicolas et al., 2022). Findings from these studies reveal a complex and sometimes inconsistent pattern. Clinically, women often report greater stress-induced craving (Elman et al., 2001), although sex differences in cue- and drug-induced craving remain equivocal (Fox et al., 2006; Moran-Santa Maria et al., 2014). Preclinical work provides mechanistic insight into these effects. Estradiol enhanced cocaine reinforcement and reinstatement, whereas progesterone and its metabolites appeared to exert protective effects (Becker et al., 2017; Larson et al., 2007; Nicolas et al., 2022; Swalve et al., 2016b). In short, reinstatement varies across the estrous cycle, and estradiol’s rapid modulation of striatal dopamine release underscores the role of ovarian hormones in relapse susceptibility (Kokane and Perrotti, 2020). Beyond sex differences, social factors also shape drug seeking. In socially housed nonhuman primates, placement within the social hierarchy reflects a continuum in which subordination corresponds to chronic social stress and dominance is associated with social and environmental enrichment (Czoty et al., 2009; Kromrey et al., 2016; Nader et al., 2012a). This continuum has strong translational value and reliably influences cocaine self-administration (Allen and Nader, 2025; Czoty et al., 2017). These observations highlight that sex and social hierarchy may influence cocaine reinforcement and point to the importance of incorporating these factors into translational models that more accurately reflect clinical relapse risk.

Thus, the goal of the present study was to investigate how cocaine and cocaethylene priming doses influenced drug-induced reinstatement in socially housed male and female cynomolgus macaques. To address additional gaps in the polysubstance use and reinstatement literature, this study also examined whether co-administration of nicotine altered reinstatement outcomes and whether sex or social rank moderated these effects. By incorporating polysubstance use, the social environment, and individual differences, this model provides a more translational framework for understanding relapse and identifying variables that moderate cocaine- and cocaethylene-induced drug seeking.

## 2 Materials and methods

### 2.1 Subjects

Twenty-two adult cynomolgus macaques (12 males, 10 females) served as subjects (Supplementary Table 1). All 22 monkeys participated in Experiment 1. Two monkeys (F-8534 and F-8535) did not complete Experiment 2 and were therefore excluded from the Experiment 2 analyses (Supplementary Table 2). All monkeys but one (M-8103) were housed in same-sex social groups of 3 or 4 and social rank was determined according to the outcomes of agonistic encounters as described previously (Morgan et al., 2000). M-8103 was individually housed throughout the study and thus, analyses that investigated social rank excluded him. In socially housed monkeys, social ranks were well-established ( >3 years) and did not change over the course of the study. For this study, the number 1- and 2-ranked monkeys were considered dominant while the number 3- and 4-ranked monkeys were classified as subordinate. This was done to maximize statistical power. Importantly, a prior paper (Morgan et al., 2000) from our laboratory analyzed the frequency of aggressive and submissive behaviors as a function of social rank and found that the 3- and 4-ranked monkeys demonstrate higher rates of submissive behaviors when compared to the 1- and 2-ranked monkeys which show more aggressive behaviors. These findings support categorizing the 2-ranked monkey as dominant and the 3- ranked monkey as subordinate rather than classifying them as intermediates in the social hierarchy. The sex and social ranks of all monkeys are shown in Supplementary Table 1. The menstrual cycle in all females was monitored daily by vaginal swabs. Studies were typically conducted 5 days per week and independent of the menstrual cycle. Prior to the start of this study, the monkeys had a chronic history ( > 1 year) of self-administering cocaine (0.001–0.1 mg/kg) under various schedules of reinforcement including a fixed-ratio (FR) schedule of reinforcement and a concurrent drug vs. food choice procedure (Allen et al., 2024a; Allen et al., 2024b; Johnson et al., 2024; Johnson et al., 2025). Most monkeys also had a history of self-administering nicotine and cocaethylene (Allen et al., 2023; Allen et al., 2024b).

All monkeys were housed in stainless-steel cages, in a temperature- and humidity-controlled room maintained on a 14-h light/10-h dark cycle (lights on between 6:00 a.m. and 8:00 p.m.). Information about diet and enrichment can be found in previously published studies (Allen et al., 2025a; Allen et al., 2025b). Animal housing, handling, and experimental protocols were performed per the 2011 National Research Council *Guidelines for the Care and Use of Mammals in Neuroscience and Behavioral Research* and were approved by the Animal Care and Use Committee of Wake Forest University.

### 2.2 Catheter implantation

For drug self-administration, each monkey was surgically implanted with a chronic indwelling intravenous catheter and subcutaneous vascular access port (VAP) under sterile conditions. Details of this surgery can be found in previously published work (Allen et al., 2023; Allen et al., 2025b). All monkeys were given at least 1 week to recover from the surgery before experimental sessions began.

### 2.3 Apparatus

All monkeys, fitted with aluminum collars, were trained to sit in a primate chair (Primate Products, Redwood City, CA). Cocaine self-administration experiments were conducted in ventilated, sound-attenuating primate chambers (1.5 × 0.76 × 0.76 m; Med Associates, St. Albans, VT). Operant responding involved breaking a beam on a photo-optic switch (Model 117-1007; Stewart Ergonomics, Inc., Furlong, PA); each chamber had two switches and associated discriminative stimuli above them. A description of the operant chamber can be found in Allen et al. (2023).

### 2.4 Drug-induced reinstatement procedure

Prior to the start of this study, monkeys were maintained under a concurrent drug vs. food choice schedule of reinforcement. Descriptions of this procedure can be found elsewhere (Allen et al., 2024b). Briefly, completion of an FR 30 on one switch produced a 1.0-g banana-flavored food pellet; completion of an FR 30 on the alternate switch produced an i.v. infusion of cocaine (0.001–0.1 mg/kg/injection). Alternating between switches before completion of the FR 30 reset the FR value. Completion of a session occurred after 30 trials or 60 min elapsed, whichever came first. The total number of trials was lowered to 15 in all female monkeys due to the monkeys leaving food pellets when 30 reinforcers were available. To establish extinction of drug responding, cocaine on the drug-associated switch was replaced with saline while the food contingency remained unchanged. Extinction sessions were conducted under the same concurrent FR schedule and responding was deemed extinguished when % Drug-associated choice decreased to 20% or lower for a minimum of three consecutive sessions. % Drug-associated choice was calculated as the proportion of total reinforcers earned on the drug-associated lever relative to the total number of reinforcers earned during the session.

Once extinction criteria were met, drug-induced reinstatement tests were conducted, typically on Tuesdays and Fridays. For most monkeys, after 2–3 reinstatement test sessions, there was a return to cocaine vs. food choice under the self-administration paradigm. These self-administration sessions used the lowest dose of cocaine that engendered > 80% drug choice and typically were conducted for 1–2 sessions and until choice was again > 80% on the drug side. Once that occurred, saline replaced cocaine on the drug-associated switch and reinstatement testing resumed when % drug-associated choice was < 20%. In Experiment 1, monkeys received an intravenous pretreatment of cocaine (0.01–0.3 mg/kg), or cocaethylene (0.03–0.3 mg/kg) 3 min before the start of the choice session. During these test sessions, saline was available on the drug-associated switch and food (one, 1.0-g banana-flavored pellet) remained available on the alternative switch under the concurrent FR schedule. Each dose of cocaine and cocaethylene was tested at least twice in pseudorandom order, with extinction (saline-pretreatment) sessions interposed between test sessions. In Experiment 2, nicotine (0.01–0.056 mg/kg) was evaluated alone and in combination with cocaine or cocaethylene. For combination tests, monkeys received nicotine + cocaine or nicotine + cocaethylene pretreatments that were mixed and delivered in the same syringe. Nicotine-alone sessions were conducted under conditions identical to reinstatement tests, with saline available on the drug-associated switch. All nicotine doses and drug combinations were tested at least twice in pseudorandom order, with extinction sessions between tests.

### 2.5 Drugs

(-)Cocaine HCl and cocaethylene fumarate were supplied by the National Institute on Drug Abuse (Bethesda, MD) and were dissolved in sterile 0.9% saline. Cocaine was expressed in the salt form and a correction was applied to the cocaethylene fumarate (dose/0.366) to compare doses across drugs. Nicotine bitartrate (Sigma-Aldrich, St. Louis, MO), was also dissolved in sterile 0.9 % saline and expressed as the salt.

### 2.6 Data analyses

#### 2.6.1 Experiment 1: comparison of cocaine- and cocaethylene-induced reinstatement

Individual-subject data are presented as means ± SD, and group data are shown as means ± SEM. The primary dependent variable was % drug-associated choice and this was defined as the proportion of total injections on the drug-associated switch relative to the proportion of stimulus deliveries associated with both the drug- and food-associated switches. Paired *t*-tests were conducted for each individual monkey to determine whether at least one dose of cocaine and cocaethylene significantly increased % drug-associated choice relative to saline (note: we describe choice for saline as “drug-associated” choice because they were choosing saline injections over food presentation).

In addition, to investigate whether cocaine- or cocaethylene-induced reinstatement varied by social rank or sex, mixed-effects ANOVAs were used to compare average % drug-associated choice following saline to those following cocaine and cocaethylene pretreatments at the dose of cocaine and cocaethylene that had the maximal effect on % drug-associated choice. As noted above, all data points were determined at least twice in pseudorandom order, and these double-determined points were averaged in all analyses. No data were excluded. The lowest cocaine and cocaethylene dose that produced significantly higher % drug-associated choice than saline was also compared across sex or social rank using mixed-effects ANOVAs to assess potential potency differences. The dose selection for cocaine and cocaethylene in all statistics was constrained a priori. Significant ANOVAs were followed by Holm–Sidak *post-hoc* pairwise comparisons. For all statistical tests, significance was set at α = 0.05, and all analyses were conducted in SPSS and Bonferroni’s correction was applied.

#### 2.6.2 Experiment 2: effect of nicotine on cocaine and cocaethylene-induced reinstatement

Individual-subject data are presented as means ± SD, and group-level data are shown as means ± SEM. The primary dependent variable was % drug-associated choice. All data points were determined at least twice in pseudorandom order, and these double-determined points were averaged in statistical analyses. No data were excluded. For each monkey, paired *t*-tests were used to assess whether at least one dose of nicotine significantly modulated cocaine- or cocaethylene-induced reinstatement. When multiple nicotine doses were tested, the dose that produced the largest change in % drug-associated choice was selected for statistical analyses. Likewise, when nicotine was tested against more than one dose of cocaine or cocaethylene, analyses used the dose of cocaine or cocaethylene that engendered the highest % drug-associated choice. In all cases, drug doses that produced floor or ceiling effects (very low or very high % drug-associated choice) were avoided to ensure sensitivity to increases or decreases in drug-induced reinstatement with nicotine pretreatments. This method for selecting which dose would be included in group statistics was constrained a priori. Individual subject analyses were not conducted in F-8557 because cocaine + nicotine pretreatments were not double-determined.

At the group level, mixed-effects ANOVAs were conducted to determine whether sex or social rank influenced the effect of nicotine pretreatment on cocaine- or cocaethylene-induced reinstatement. As with the individual-subject analyses, the nicotine dose showing the largest behavioral effect was used. A separate mixed-effects ANOVA evaluated whether nicotine pretreatment alone increased % drug-associated choice relative to saline baselines as a function of sex and social rank. For this analysis, monkeys M-8558 and F-8557 were excluded because nicotine alone was not tested (Supplementary Table 2). Significant ANOVAs were followed by Holm–Sidak *post hoc* comparisons. Statistical significance was set at α = 0.05. All analyses were conducted in SPSS, and Bonferroni correction was applied where appropriate.

In the primary analyses, we selected the nicotine dose that produced the largest behavioral effect on cocaine- and cocaethylene-induced reinstatement to maximize statistical power, given substantial intersubject variability in the dose that was most effective. Because this selection approach potentially inflated the observed effects, we conducted follow-up analyses. Specifically, we used Repeated-Measures ANOVA to test whether nicotine’s effect on the percentage of drug-associated choice after cocaine and cocaethylene pretreatments differed by nicotine dose (0.01, 0.03, or 0.056 mg/kg). Sex and rank were included as factors of interest. Statistical significance was set at α = 0.05. All analyses were performed in SPSS.

## 3 Results

### 3.1 Experiment 1: comparison of cocaine and cocaethylene-induced reinstatement

Analyses of individual-subject data demonstrated that all monkeys had at least one dose of cocaine that increased % drug-associated choice relative to saline (Supplementary Table 1 and Supplementary Figures 1A,B). All monkeys but one (M-8103) had at least one dose of cocaethylene that significantly increased % drug-associated choice relative to saline (Supplementary Figures 1A,B). The number of total number of trials completed for each individual monkey can be found in Supplementary Figures 2A,B. In most monkeys, most trials were completed across the full range of doses tested; however, in some individuals (F-8555, M-8559, M-8506), the highest doses of cocaine and/or cocaethylene tested produced decreases in the number of trials completed relative to saline (*p* < 0.05) (Supplementary Figures 2A,B).

**FIGURE 1:**
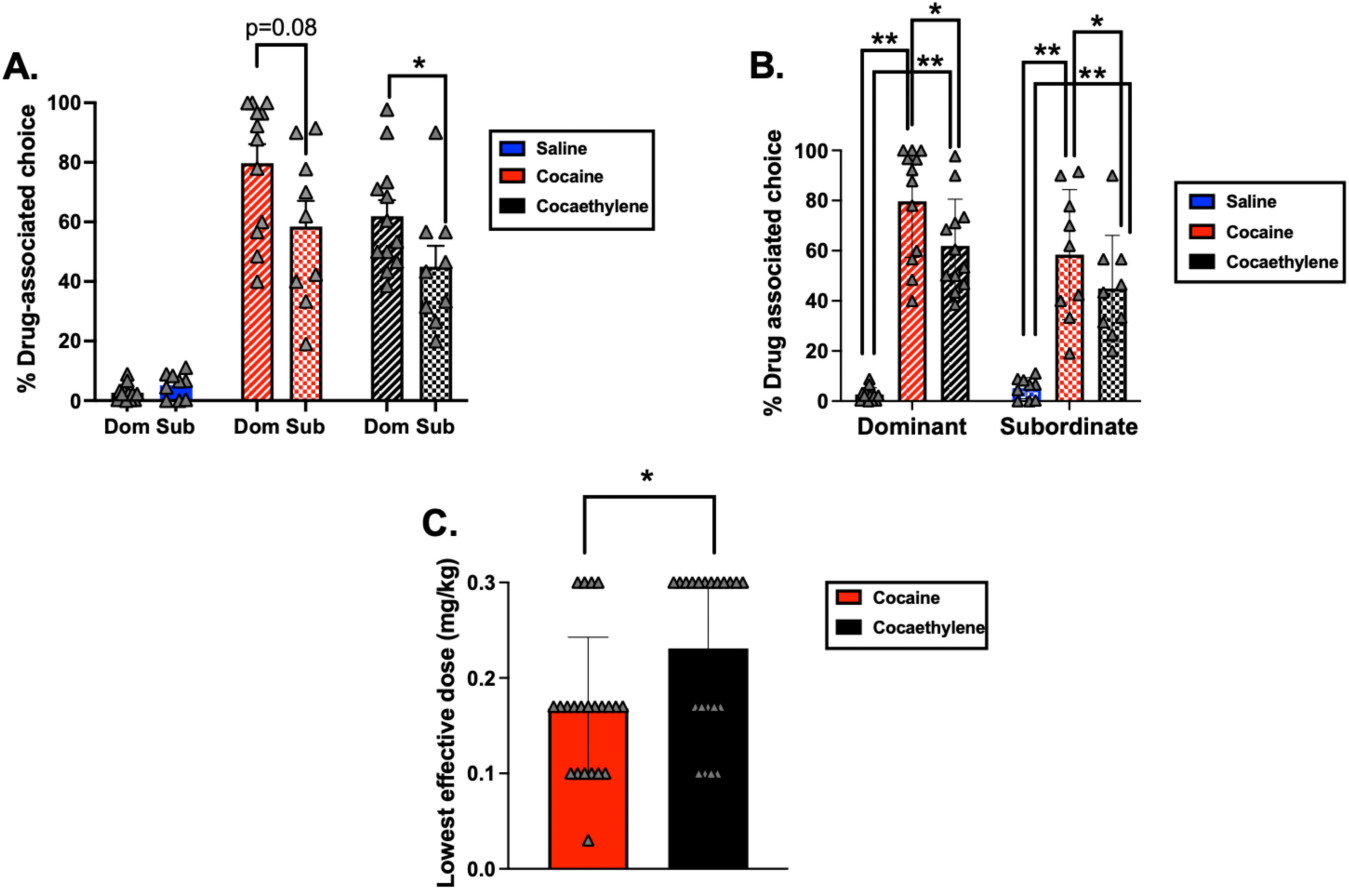
Percent drug-associated choice (% choice) at baseline and following cocaine or cocaethylene pretreatments in dominant and subordinate monkeys (*n* = 7 dominant males, *n* = 4 subordinate males, *n* = 5 dominant females, *n* = 5 subordinate females). **(A,B)** Present the same dataset in two formats to clarify relationships between social rank. **(A)** % drug-associated choice separated by rank across pretreatment conditions. **(B)** Within-rank comparison of % drug-associated choice across pretreatment conditions. **(C)** Lowest effective doses of cocaine and cocaethylene that produced significant increases in % drug-associated choice relative to saline. Data represent the mean ± the standard error of the mean. *p < 0.05; **p < 0.001. Individual-subject data are shown in gray triangles.

Using the cocaine and cocaethylene dose that had the greatest effect on % drug-associated choice (Supplementary Figures 1A,B), analyses at the group-level found a significant interaction between drug condition (% drug-associated choice when saline, cocaine or cocaethylene were given as pretreatments) and the social rank of the monkey [*F*(2, 17) = 3.89, *p* = 0.030]. *Post-hoc* tests revealed that there were no significant differences in % drug-associated choice between dominant and subordinate monkeys when saline was given as a pretreatment (*p* > 0.05) (Figure 1A). In both dominant and subordinate monkeys, both cocaine and cocaethylene pretreatments engendered % drug-associated choice higher than saline baselines (*p* < 0.001 for all). Moreover, comparing maximal effects in both dominant and subordinate monkeys, cocaine pretreatments resulted in higher % drug-associated choice than cocaethylene (*p* = 0.006, *p* = 0.023, respectively) (Figure 1B). While differences in dominant monkeys and subordinate monkeys did not reach statistical significance when comparing % drug-associated choice following cocaine pretreatments (*p* = 0.084), dominant monkeys had significantly higher % drug-associated choice following cocaethylene pretreatments when compared to subordinate monkeys (*p* = 0.042) (Figure 1A).

As it relates to potency comparisons, analyses of the lowest cocaine and cocaethylene doses that produced significantly higher % drug-associated choice than saline pretreatments (Supplementary Table 1 and Supplementary Figures 1A,B) found a significant main effect of drug condition [cocaine dose, cocaethylene dose; *F*(1, 17) = 12.87, *p* = 0.002] such that cocaine significantly increased % drug-associated choice relative to saline at lower doses than cocaethylene (Figure 1C). This was independent of sex or social rank. One important note is that in some monkeys, the lowest dose of cocaine or cocaethylene tested resulted in significantly higher % drug-associated choice than saline (Supplementary Figures 1A,B) and thus, drug potency may be underestimated in some animals. However, when these animals (F-8581, F-8535, F-8551, F-8548, M-8180, and M-8677) were excluded from the above analysis, cocaine still significantly increased % drug-associated choice relative to saline at lower doses than cocaethylene [*F*(1, 12) = 14.49, *p* = 0.002].

### 3.2 Experiment 2: effect of nicotine on cocaine and cocaethylene-induced reinstatement

Analyses of individual-subject data (Supplementary Table 2) showed that nicotine alone reinstated responding in 7 monkeys (see below). Among female monkeys, adding nicotine to cocaine significantly increased % drug-associated choice relative to that dose of cocaine alone in 2 of 3 dominant and 4 of 5 subordinate monkeys (Supplementary Figure 3A); nicotine did not significantly alter cocaine-induced reinstatement in the two remaining females. Among dominant males, nicotine significantly potentiated cocaine-induced reinstatement in 1 of 7 monkeys and significantly reduced reinstatement in 4 of 7 monkeys, with no significant effect in the remaining 2 monkeys. In subordinate males, nicotine significantly reduced cocaine-induced reinstatement in 1 of 4 monkeys and had no significant effect in the other 3 animals. Nicotine also reduced cocaine-induced reinstatement in the individually housed male, M-8103 (Supplementary Figure 3B).

For cocaethylene-induced reinstatement, individual-subject data showed that adding nicotine increased reinstatement in 2 of 3 dominant and 2 of 4 subordinate females and decreased reinstatement in 1 of 4 subordinate females (Supplementary Figure 4A); nicotine had no significant effect in the remaining females. Among males, there was substantial variability when nicotine was added to cocaethylene (Supplementary Figure 4B): nicotine reduced cocaethylene-induced reinstatement in 1 of 7 dominant and 2 of 4 subordinate males, increased reinstatement in 1 of 7 dominant males, and had no significant effect in the remaining males including M-8103.

The total number of trials completed by each individual monkey is shown in Supplementary Figures 5A,B, 6A,B. For most monkeys, trials were completed across the full range of doses tested for cocaine, cocaethylene, nicotine, cocaine + nicotine, and cocaethylene + nicotine. However, in some individuals (F-8555, M-8559, M-8506, F-8551), the highest doses of cocaine and/or cocaethylene produced fewer completed trials relative to saline (*p* < 0.05; Supplementary Figures 5A,B, 6A,B). In addition, in two monkeys (F-8551, M-8502; Supplementary Figures 5A,B), adding at least one dose of nicotine to cocaine significantly reduced the total number of trials completed (*p* < 0.05). Similarly, in three monkeys (F-8555, F-8551, M-8502; Supplementary Figures 6A,B), adding nicotine to cocaethylene significantly reduced the total number of trials completed (*p* < 0.05).

**FIGURE 2:**
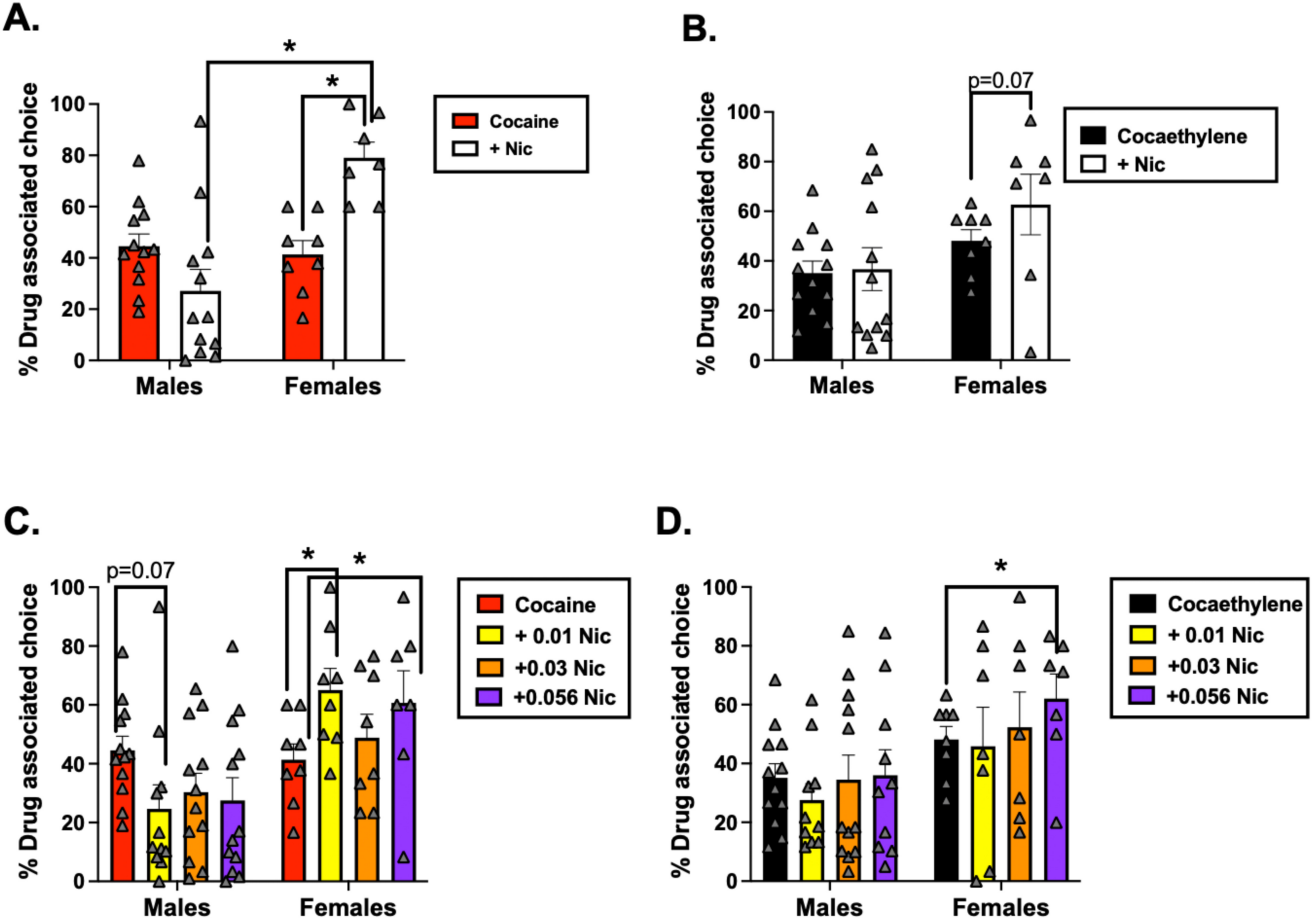
% Drug-associated choice following pretreatment with **(A)** cocaine alone versus cocaine + nicotine and **(B)** cocaethylene alone versus cocaethylene + nicotine in male and female monkeys. In panels **A,B**, the nicotine dose that produced the largest change in % drug-associated choice is shown. **(C,D)** Dose-dependent effects of nicotine on **(C)** cocaine- and **(D)** cocaethylene-induced reinstatement, with data plotted separately for each nicotine dose (0.01, 0.03, and 0.056 mg/kg). Group sizes were: dominant males (*n* = 7), subordinate males (*n* = 4), dominant females (*n* = 5), and subordinate females (*n* = 4). Bars represent mean ± SEM. *p < 0.05; exact *p*-values are shown where indicated. Individual-subject data are shown in gray triangles.

Using the nicotine dose that produced the greatest change in % drug-associated choice, group-level analyses showed a significant interaction between drug condition (% drug-associated choice with cocaine alone vs. cocaine + nicotine) and sex [*F*(1, 15) = 15.64, *p* = 0.001]. *Post hoc* tests indicated that males and females did not differ in % drug-associated choice when cocaine was administered alone (*p* = 0.583). However, when cocaine was co-administered with nicotine, females showed a significantly higher % drug-associated choice than males (*p* = 0.001). Further analyses showed that adding nicotine significantly increased % drug-associated choice in females (cocaine alone vs. cocaine + nicotine: *p* = 0.002), but not in males (*p* = 0.107) (Figure 2A). Social rank was not a significant factor in this model (*p* > 0.05). No significant main effect or interactions were observed for cocaethylene-induced reinstatement when nicotine was added (all *p* > 0.05) (Figure 2B). However, among female monkeys, % drug-associated choice was marginally higher with cocaethylene + nicotine when compared to cocaethylene alone (*p* = 0.073). This was not present in male monkeys (*p* = 0.908).

**FIGURE 3:**
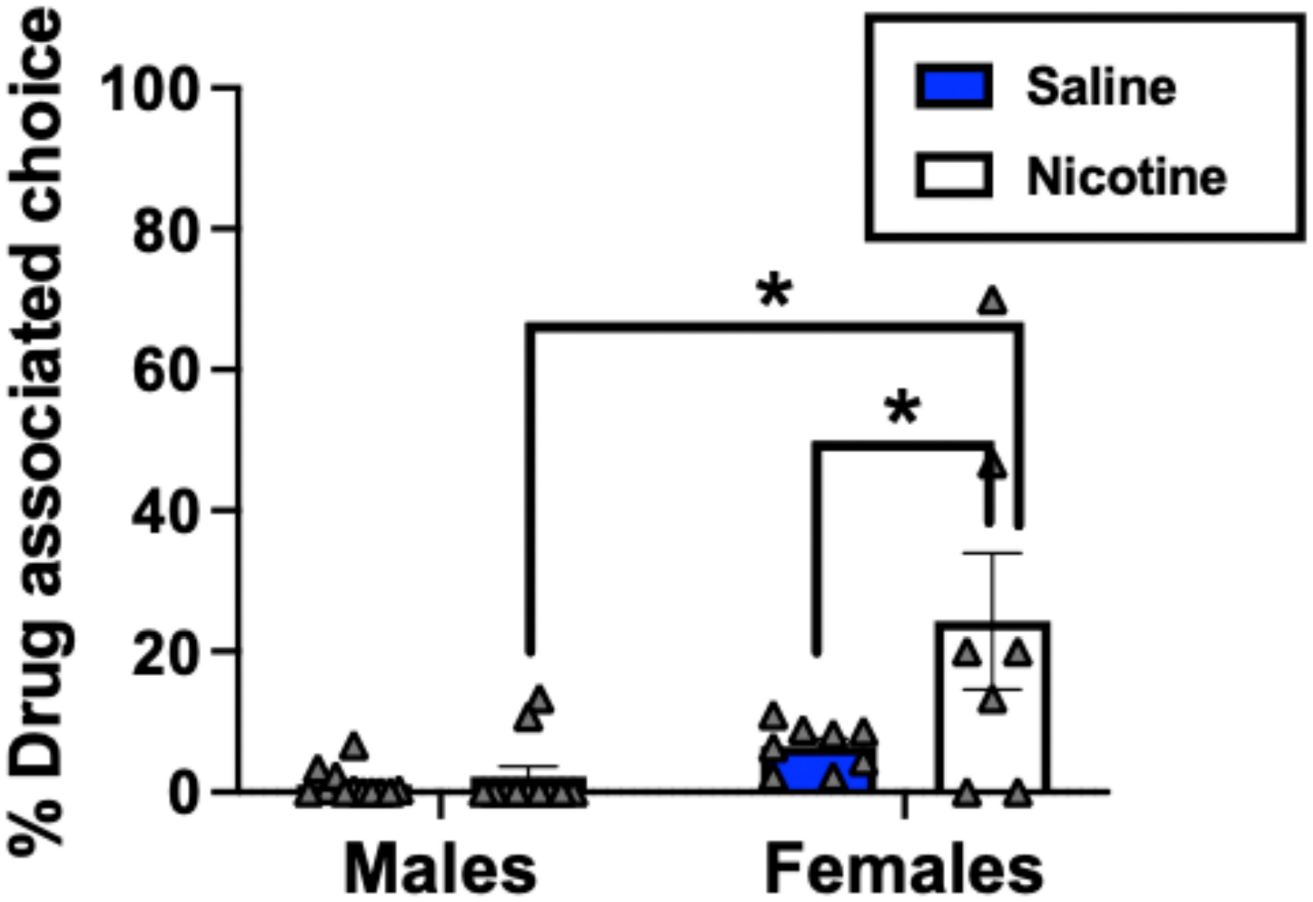
Percent drug-associated choice at baseline and following nicotine pretreatments in dominant and subordinate monkeys of both sexes (*n* = 7 dominant males, *n* = 4 subordinate males, *n* = 5 dominant females, *n* = 5 subordinate females). For each drug, data are shown at the nicotine dose that produced the largest effect. Bars represent mean ± SEM. *p < 0.05. Individual-subject data are shown in gray triangles.

Analyses examining whether nicotine alone produced drug-induced reinstatement relative to saline revealed an interaction between drug condition (nicotine pretreatments vs. saline baselines on % drug-associated choice) and sex [*F*(1, 13) = 5.20, *p* = 0.040]. Nicotine pretreatments significantly increased % drug-associated choice relative to saline only in female monkeys (*p* = 0.009); nicotine did not significantly produce drug-induced reinstatement in males and social rank was not significant (*p* > 0.05). Furthermore, when nicotine was given as a pretreatment, females showed a significantly higher % drug-associated choice than males (*p* = 0.025) (Figure 3).

While the above analyses included the dose of nicotine that had the greatest effect on drug-induced reinstatement, multiple nicotine doses were tested in combination with cocaine and cocaethylene (Supplementary Figures 3, 4). Thus, we used Repeated-Measures ANOVAs to determine whether nicotine’s effect on % drug-associated choice following cocaine and cocaethylene pretreatments varied as a function of nicotine dose (0.01–0.056 mg/kg). For cocaine-induced reinstatement, there was a significant sex × dose interaction [*F*(1, 14) = 4.84, *p* = 0.045]. Adding 0.01 mg/kg nicotine to cocaine produced a marginal decrease in % drug-associated choice in males (*p* = 0.072) but a significant increase in females (*p* = 0.044). In males, neither 0.03 nor 0.056 mg/kg nicotine significantly altered % drug-associated choice following cocaine pretreatment. In females, 0.056 mg/kg nicotine significantly increased % drug-associated choice (*p* = 0.024), whereas 0.03 mg/kg nicotine had no effect on cocaine-induced reinstatement (Figure 2C). For cocaethylene-induced reinstatement, a similar pattern emerged, with a marginal sex × dose interaction [*F*(1, 13) = 2.57, *p* = 0.068]. Nicotine did not significantly alter cocaethylene-induced reinstatement in males at any dose. In females, however, 0.056 mg/kg nicotine significantly increased % drug-associated choice following cocaethylene pretreatment (*p* = 0.047); no other nicotine doses reached significance (Figure 2D). Social rank had no effect in either analysis (*p* > 0.05).

## 4 Discussion

The present study examined how sex, social rank, and nicotine exposure interact to influence cocaine- and cocaethylene-induced reinstatement under a food vs. drug choice procedure in socially housed cynomolgus monkeys. Across both sexes and social ranks, pretreatments with cocaine and cocaethylene reliably increased drug-associated choice relative to saline baselines, with dominant monkeys showing greater reinstatement than subordinates for cocaethylene and marginally greater reinstatement for cocaine. Cocaine was more potent than cocaethylene, producing significant reinstatement at lower doses. When nicotine was added, its effects were influenced by sex: nicotine co-administration increased cocaine-induced reinstatement in females, regardless of social rank. However, it did not impact cocaine-induced reinstatement in males and often reduced the effects of cocaine-induced reinstatement at the individual-subject level. Nicotine had more variable and generally weaker effects on cocaethylene-induced reinstatement, with only a trend toward increasing cocaethylene-induced reinstatement in females. Finally, nicotine alone reinstated drug-associated responding only in females. Together, these findings suggest that common polysubstance combinations (cocaine, alcohol/cocaethylene, and nicotine), the social environment, and biological sex interact to determine relapse-related behavior. Furthermore, they highlight heightened sensitivity to the effects of nicotine in females.

This study extended the comparison of cocaine and cocaethylene to a reinstatement model, where cocaine was more potent than cocaethylene and cocaine tended to result in greater reinstatement. Earlier research comparing the behavioral potency of cocaine and cocaethylene noted differences depending on the experimental paradigm. For example, Katz et al. (1992) found that cocaine was 4-times more potent than cocaethylene in increasing operant responding by mice and 5-times more potent than cocaethylene in cocaine discrimination studies in rats and squirrel monkeys. In contrast, Allen et al. (2023) reported that cocaine and cocaethylene were equipotent in monkeys responding under a progressive-ratio and concurrent choice contingencies. It has been speculated that reinstatement models are similar to studies involving drug discrimination (Katz and Higgins, 2003). The present study supports this premise by demonstrating that cocaine is also more potent than cocaethylene in a model of drug-induced reinstatement.

In addition to potency differences between cocaine and cocaethylene, these experiments demonstrated that social rank significantly influenced drug-induced reinstatement when examining the dose of drug that had the greatest effect on % drug-associated choice. In this study, dominant monkeys exhibited significantly higher % drug-associated choice following cocaethylene pretreatments and marginally higher % drug-associated choice following cocaine exposure when compared to their subordinate counterparts. This social rank-dependent difference underscores the importance of social context in modulating drug-seeking behavior. Previous research has demonstrated that chronic social subordination in monkeys is a well-validated model of social stress (Kaplan et al., 1986; Kaplan et al., 1999) and can increase susceptibility to a host of diseases including atherosclerosis (Kaplan et al., 1982), upper respiratory infection (Cohen et al., 1997), and reproductive dysfunction (Kaplan et al., 2010). Subordinate monkeys also exhibit heavier adrenal glands when compared to dominants (Shively and Kaplan, 1984), consistent with the view that social rank alters hypothalamic–pituitary–adrenal (HPA) axis physiology, a key regulator of stress responses (Henry and Stephens, 1977).

Prior work in our laboratory has shown that after long-term stable social housing when compared to dominant monkeys, subordinate males (Morgan et al., 2002) and females were more vulnerable to cocaine reinforcement on an acquisition procedure (Johnson et al., 2024; Nader et al., 2012b). However, a similar relationship between social rank and sensitivity to cocaine was not seen in this study. Instead, this study found that dominant monkeys had greater drug-associated choice with both cocaine and cocaethylene pretreatments. Previous work has shown that while subordinate monkeys are more vulnerable to cocaine initially, after long-term cocaine self-administration, response rates and cocaine intakes are no longer different between dominant and subordinate male monkeys who self-administer cocaine alone (Czoty et al., 2004). Hence, it is possible that the factors influencing initial vulnerability to cocaine reinforcement are not the same as those that influence other facets of drug use such as the reinstatement of drug-seeking. Subordination and chronic social stress have been linked to reduced basal dopaminergic tone and lower striatal dopamine D2/D3 receptor availability (Morgan et al., 2002; Nader et al., 2012b), which are neuroadaptations that promote initial vulnerability to cocaine acquisition (Morgan et al., 2002). On the other hand, social dominance has been associated with increased D2/D3 availability and lower vulnerability to cocaine reinforcement (Johnson et al., 2024; Morgan et al., 2002). However, under a reinstatement paradigm, higher D2/D3 receptor availability in dominant monkeys could plausibly enhance the salience of the interoceptive cues of cocaine and of drug-paired stimuli, thereby increasing the efficacy of pharmacological primes to reactivate drug-seeking in dominant animals (Nader and Czoty, 2005; Wolff and Saunders, 2023). In short, greater drug-induced reinstatement in dominant monkeys may suggest context-dependent differences in stress-mediated initiation (acquisition) and cue- or interoception-mediated relapse (reinstatement). The behavioral, neurochemical and neuropharmacological mechanisms underlying these social rank-related differences in drug-induced reinstatement deserve further investigation.

While social rank influenced cocaine- and cocaethylene-induced reinstatement, the addition of nicotine shifted the primary moderating factor to sex. Only female monkeys showed increased % choice of the drug-associated lever when nicotine was added to cocaine, and a similar, although non-significant, pattern emerged for cocaethylene. In follow-up analyses that included all nicotine doses tested, statistical analyses confirmed that the nicotine-related effects observed in the primary analyses were not an artifact of selecting the dose with the largest behavioral impact. Specifically, when comparing monkeys with the same nicotine dose, nicotine’s modulation of cocaine- and cocaethylene-induced reinstatement remained sex-dependent. Importantly, complete nicotine dose-response curves were determined with cocaine and cocaethylene and for some monkeys, lower nicotine doses resulted in greater reinstatement than higher nicotine doses. While the reason for these differences in sensitivity are not apparent, these data highlight important individual differences in the behavioral effects of nicotine. Furthermore, nicotine alone produced drug-induced reinstatement only in females, indicating that females may be more sensitive to the effects of nicotine in general and to the effects of nicotine on cocaine- and cocaethylene-induced reinstatement. These observations align with prior clinical and preclinical work showing sex differences in nicotine sensitivity. For instance, one clinical study found that women were more sensitive to the subjective effects of intravenous nicotine and reported enhanced ratings of “drug strength” and “head rush” when compared to men (Sofuoglu and Mooney, 2009). In rodents, females acquired nicotine reinforcement more rapidly under an FR schedule (Donny et al., 2000) and had higher peak breakpoints under a PR schedule when compared to males (Li et al., 2014).

However, studies using reinstatement procedures in rodents have yielded mixed results, with some reporting no sex differences (Feltenstein et al., 2012), others finding greater nicotine-induced reinstatement in males (Swalve et al., 2016a) and still others reporting enhanced nicotine-induced reinstatement in females (Kilty et al., 2024). Our data add to this literature by showing that nicotine resulted in drug-induced reinstatement only in females. Furthermore, the premise that females are more sensitive to the effects of nicotine when combined with cocaine is also consistent with prior work from our laboratory, which showed that the addition of nicotine to cocaine potentiated cocaine choice to a greater extent in females compared to males, regardless of social rank (Allen et al., 2024b). Future studies will be needed to determine how nicotine and cocaethylene interact under self-administration procedures and how those interactions relate to the current reinstatement findings.

In the present study, only females showed significant reinstatement with nicotine alone, and one possibility to explain this finding is that the increase in cocaine- and cocaethylene-induced reinstatement with nicotine co-administration reflects an additive effect of the two drugs. However, this interpretation has an important caveat: in several monkeys, nicotine alone did not increase drug-associated responding, yet it still significantly increased % drug-associated choice when combined with cocaine or cocaethylene. This pattern suggests that nicotine’s potentiation of drug-induced reinstatement is more complex than simple additivity. Because many females exhibited irregular menstrual cycles during this study, we could not assess its impact on the effect of nicotine on drug-induced reinstatement. Therefore, it remains possible that nicotine’s effects varied across cycle phase. The menstrual cycle irregularities noted within our cohort of female macaques could be due to a variety of factors. It is well established in preclinical and clinical studies that stressors, including social stress, can alter menstrual cycles in women (Barsom et al., 2004) and female animals (Bethea et al., 2008). Along with social stressors, chronic cocaine use has also been shown to alter menstrual cycles in preclinical and clinical studies (Mello and Mendelson, 1997; Mello et al., 1997). In fact, one study found that cocaine self-administration in rhesus monkeys was related to low progesterone levels and luteal phase dysfunction. Moreover, it resulted in amenorrhea which persisted even when cocaine self-administration was discontinued (Mello et al., 1997). In one study from our laboratory, we examined whether acquisition of cocaine self-administration under a fixed-ratio schedule was influenced by menstrual cycle and did not find differences (Nader et al., 2012b). It is certainly the case that the role of menstrual cycle on measures of the behavioral and reinforcing effects of cocaine will be influenced by experimental parameters, social factors and drug history.

Although the mechanisms behind the irregular menstrual cycles are not fully understood, one study (Mello et al., 1997) hypothesized that it could be due to changes in baseline levels of anterior pituitary hormones that regulate menstrual cycles such as luteinizing hormone (LH), follicle-stimulating hormone (FSH), adrenocorticotropic hormone (ACTH), and prolactin. One important point is that several studies have shown that progesterone may decrease the reinforcing effects of numerous drugs including nicotine (Lynch and Sofuoglu, 2010; Quinones-Jenab and Jenab, 2010). Thus, one possibility is that chronic cocaine self-administration in this cohort of female monkeys resulted in prolonged progesterone depletion which potentiated nicotine + drug-induced reinstatement. Future work should explicitly test whether variability in nicotine sensitivity in monkeys is related to menstrual cycle phase, hormonal differences, and/or other neurobiological differences between males and females.

Although the reinstatement paradigm has been used for decades as a preclinical model of relapse, it has important limitations. Perhaps most notably, many preclinical reinstatement procedures show limited functional equivalence, and in some cases even negative associations, with relapse outcomes in the clinical literature (Katz and Higgins, 2003). One key issue is that relapse outside the laboratory typically occurs following drug exposure during ongoing access, rather than after experimenter-delivered “pretreatments” [or what Katz and Higgins (62) referred to as an “initiator” of relapse]. Despite these limitations, extending reinstatement models to a choice procedure, where behavior is not extinguished but instead reallocated to an alternative is a meaningful step toward improving translational relevance.

A second limitation, particularly relevant to comparisons of cocaine and cocaethylene potency, is the monkeys’ drug histories. All animals had substantially longer cocaine self-administration histories than cocaethylene histories prior to this experiment. This matters because saline was delivered on the same operant switch and paired with the same discriminative stimuli as the drug condition, and those cues were likely more strongly associated with cocaine than cocaethylene. Given this, the finding that cocaine was more potent than cocaethylene in inducing drug-induced reinstatement should be interpreted in the context of this important caveat. Future studies should evaluate the role that drug history plays in modifying the variables examined in this study. Moreover, because cocaine was more potent that cocaethylene, it is possible that cocaine had a greater maximal effect on % drug-associated choice than cocaethylene because high enough doses of cocaethylene were not tested. This is an important limitation to consider when interpreting the findings of this study.

Another consideration is that in some monkeys, the addition of nicotine to cocaine or cocaethylene reduced the total number of trials completed, suggesting potential rate-decreasing effects at higher dose combinations. However, in the primary group-level analyses (Figures 2A,B), the cocaine + nicotine and cocaethylene + nicotine doses included were rarely those that produced decreases in total trials. In fact, for the cocaethylene + nicotine analysis, only data from F-8555 were included beyond the point at which trial completion declined (Supplementary Figure 6A), and for the cocaine + nicotine analysis, only data from F-8551 were included at that point in the dose–response curve (Supplementary Figure 5A). Thus, rate-decreasing effects of these combinations likely had minimal impact on the group-level statistics reported. Finally, our model differs from typical human routes of self-administration which impacts drug pharmacokinetics. Cocaethylene is a metabolite formed in the liver when alcohol is consumed prior to cocaine use (Harris et al., 2003; Landry, 1992). In this study, cocaethylene was available intravenously, which may produce neuropharmacological effects that differ from the *in vivo* formation of cocaethylene. The same concern applies to nicotine: in humans it is most commonly inhaled and can be taken sequentially with cocaine or cocaine plus alcohol rather than simultaneously. By not modeling the route and temporal dynamics of nicotine or cocaine + alcohol self-administration during co-use, our procedure may not capture all clinically relevant features of polysubstance use.

Despite these limitations, the present design incorporated key variables, including sex, social rank and polysubstance use, and generated several testable hypotheses. Most notably, the effects of nicotine on cocaine- and cocaethylene-induced reinstatement differed by sex. Clinically, nicotine use is not routinely considered in trials for CUD, and smokers and non-smokers are often not analyzed separately (Anderson et al., 2009; Dackis et al., 2012; Kampman, 2019). Our findings suggest that continued nicotine exposure may be especially consequential for women with CUD and should be considered when designing and interpreting treatment studies. Importantly, while several studies suggest that alcohol use may reduce the efficacy of treatments for CUD (Anderson et al., 2009; Rough et al., 2025a), the impact of continued alcohol use on relapse probability for CUD has not been systematically investigated. This remains an important direction for future work. Overall, these data highlight the translational value of incorporating common polysubstance-use patterns, sex, and environmental variables into preclinical models to better capture factors that influence treatment outcomes for CUD.

## Data Availability

The original contributions presented in this study are included in this article/Supplementary material, further inquiries can be directed to the corresponding author.
