# Supplementary material for: Sex, social rank, and nicotine co-administration shape cocaine- and cocaethylene-induced reinstatement in monkeys
Source: Front Behav Neurosci. 2026 Feb 18;20:1770940. doi: 10.3389/fnbeh.2026.1770940 (PMC12957084; doi:10.3389/fnbeh.2026.1770940)

**Supplemental File**

**Sex, social rank, and nicotine co-administration shape cocaine- and cocaethylene-induced reinstatement in monkeys**

Brianna F. Roberts, Mia A. Clark, Michael A. Nader and Mia I. Rough

Department of Translational Neuroscience, Wake Forest University School of Medicine, Winston-Salem, NC 27157-1083 USA

**Supplemental Table 1: Individual-Subject Characteristics Associated with Cocaine- and Cocaethylene-Induced Reinstatement.**

| **Monkey** | **Sex** | **Rank** | **Food vs. Saline (% drug-associated choice)** | **Reinstatement at the cocaine dose that had the largest effect (% drug-associated choice)** | **Lowest reinstatement cocaine dose (mg/kg)** | **Reinstatement at the cocaethylene dose that had the largest effect (% drug-associated choice)** | **Lowest reinstatement cocaethylene dose (mg/kg)** |
| --- | --- | --- | --- | --- | --- | --- | --- |
| M-8180 | Male | Dom | 0.33 | 48.50 | 0.1 | 38.5 | 0.3 |
| M-8503 | Male | Dom | 0.33 | 96.50 | 0.17 | 90 | 0.17 |
| M-8502 | Male | Dom | 3.33 | 78 | 0.1 | 68.50 | 0.17 |
| M-8559 | Male | Dom | 3.33 | 100 | 0.17 | 60.5 | 0.3 |
| M-8558 | Male | Dom | 0.33 | 92.22 | 0.17 | 46.67 | 0.17 |
| M-8504 | Male | Dom | 0.00 | 88.98 | 0.3 | 53.33 | 0.3 |
| M-8506 | Male | Dom | 2.33 | 87.78 | 0.3 | 73.4 | 0.3 |
| M-8103 | Male | -- | 0.00 | 36.66 | 0.17 | 9.11 | -- |
| M-8562 | Male | Sub | 0.00 | 19 | 0.3 | 20 | 0.3 |
| M-7962 | Male | Sub | 0 | 62 | 0.17 | 26.5 | 0.3 |
| M-8677 | Male | Sub | 6.67 | 91.50 | 0.17 | 46.50 | 0.1 |
| M-8507 | Male | Sub | 0.33 | 42.33 | 0.3 | 31.5 | 0.3 |
| F-8531 | Female | Dom | 2.22 | 59.99 | 0.1 | 43.35 | 0.1 |
| F-8537 | Female | Dom | 8.89 | 96.67 | 0.17 | 71.11 | 0.17 |
| F-8555 | Female | Dom | 2.22 | 100 | 0.1 | 97.77 | 0.1 |
| F-8535 | Female | Dom | 2.22 | 40 | 0.1 | 50 | 0.3 |
| F-8534 | Female | Dom | 6.67 | 56.67 | 0.17 | 50 | 0.3 |
| F-8648 | Female | Sub | 6.56 | 40 | 0.17 | 56.67 | 0.3 |
| F-8557 | Female | Sub | 8.87 | 70 | 0.17 | 43.35 | 0.3 |
| F-8678 | Female | Sub | 11.11 | 77.78 | 0.17 | 56.67 | 0.3 |
| F-8551 | Female | Sub | 4.44 | 33.34 | 0.1 | 33.34 | 0.17 |
| F-8548 | Female | Sub | 8.33 | 90 | 0.03 | 90 | 0.1 |

**Supplemental Table 2: Individual Subject Characteristics Associated with Effects of Nicotine on Cocaine- and Cocaethylene-Induced Reinstatement.**

| **Monkey** | **Rank** | **Food vs. Saline (% drug-associated choice)** | **Cocaine-induced reinstatement (% drug-associated choice)** | **Cocaine + Nicotine (% drug associated choice)** | **Cocaethylene-induced reinstatement (% drug-associated choice)** | **Cocaethylene + Nicotine (% drug-associated choice** | **Nicotine-induced reinstatement (% drug-associated choice)** |
| --- | --- | --- | --- | --- | --- | --- | --- |
| M-8180 | Dom | 0.33 | 41.50 | 93.34 | 38.50 | 85.00 | 0 |
| M-8503 | Dom | 0.33 | 45.00 | 6.67 | 37.00 | 16.67 | 0 |
| M-8502 | Dom | 3.33 | 78.00 | 32.12 | 68.50 | 76.68 | 13.33 |
| M-8559 | Dom | 3.33 | 57.00 | 3.34 | 26.65 | 13.34 | 0 |
| M-8558 | Dom | 0.33 | 43.34 | 16.67 | 46.67 | 73.33 | -- |
| M-8504 | Dom | 0.00 | 31.67 | 8.33 | 53.34 | 33.33 | 0 |
| M-8506 | Dom | 2.33 | 23.33 | 42.28 | 14.67 | 10.23 | 0 |
| M-8103 | -- | 0.00 | 36.67 | 1.67 | 11.65 | 13.34 | 0 |
| M-8562 | Sub | 0.00 | 19.00 | .00 | 20.00 | 5.00 | 0 |
| M-7962 | Sub | 0 | 62.00 | 38.89 | 26.50 | 41.67 | 0 |
| M-8677 | Sub | 6.67 | 54.66 | 65.56 | 46.50 | 61.67 | 0 |
| M-8507 | Sub | 0 | 42.33 | 17.04 | 31.50 | 10.00 | 10.71 |
| F-8531 | Dom | 2.22 | 46.67 | 96.66 | 43.34 | 80.00 | 20.00 |
| F-8537 | Dom | 8.89 | 46.67 | 76.67 | 56.67 | 73.34 | 46.67 |
| F-8555 | Dom | 2.22 | 16.67 | 76.67 | 27.62 | 71.25 | 20.00 |
| F-8648 | Sub | 6.56 | 36.56 | 60.00 | 56.67 | 43.33 | 0 |
| F-8557 | Sub | 8.87 | 60.00 | 73.33 | -- | -- | -- |
| F-8678 | Sub | 11.11 | 60.00 | 86.67 | 56.67 | 80.00 | 70 |
| F-8551 | Sub | 4.44 | 26.67 | 100.00 | 33.34 | 3.34 | 0 |
| F-8548 | Sub | 8.33 | 37.78 | 60.00 | 63.34 | 96.67 | 13.33 |

**Supplemental Figures**

**
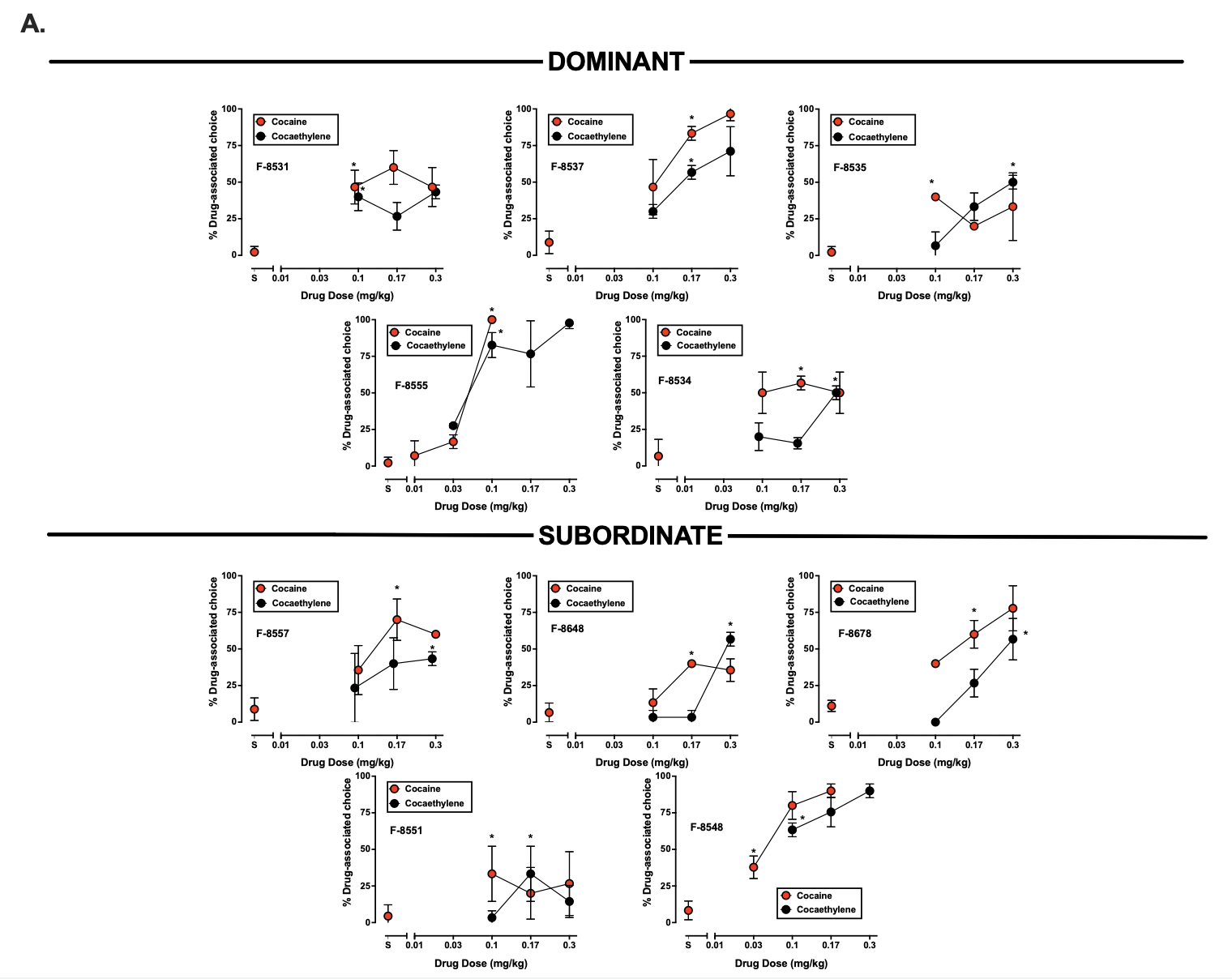
Supplemental Figure 1:** Percent drug-associated lever choice as a function of cocaine or cocaethylene dose given as a pretreatment in dominant (top panel) and subordinate (bottom panel) female (**A**) and male (**B**) monkeys. Each point is the mean ± S.D. of at least 2 sessions where a dose of cocaine or cocaethylene was administered prior to saline self-administration sessions. Asterisks depict lowest cocaine or cocaethylene dose at which % drug-associated choice were significantly higher than saline.

**
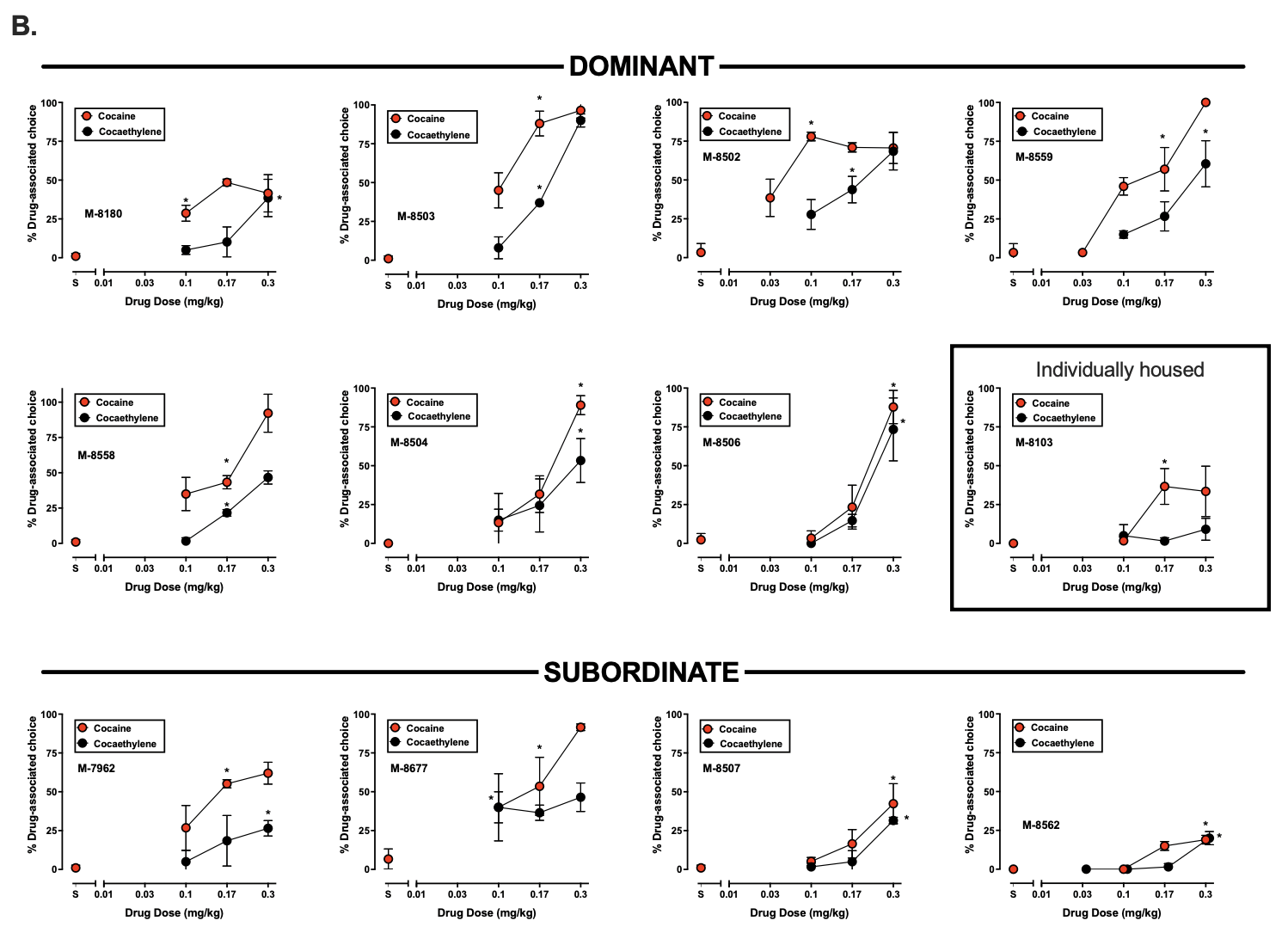
**

**Supplemental Figure 2:** Number of total trials completed as a function of cocaine or cocaethylene dose given as a pretreatment in dominant (top panel) and subordinate (bottom panel) female (**A**) and male (**B**) monkeys. Each point is the mean ± S.D. of at least 2 sessions where a dose of cocaine or cocaethylene was administered prior to saline self-administration sessions. Note differences in ordinate in males and females.


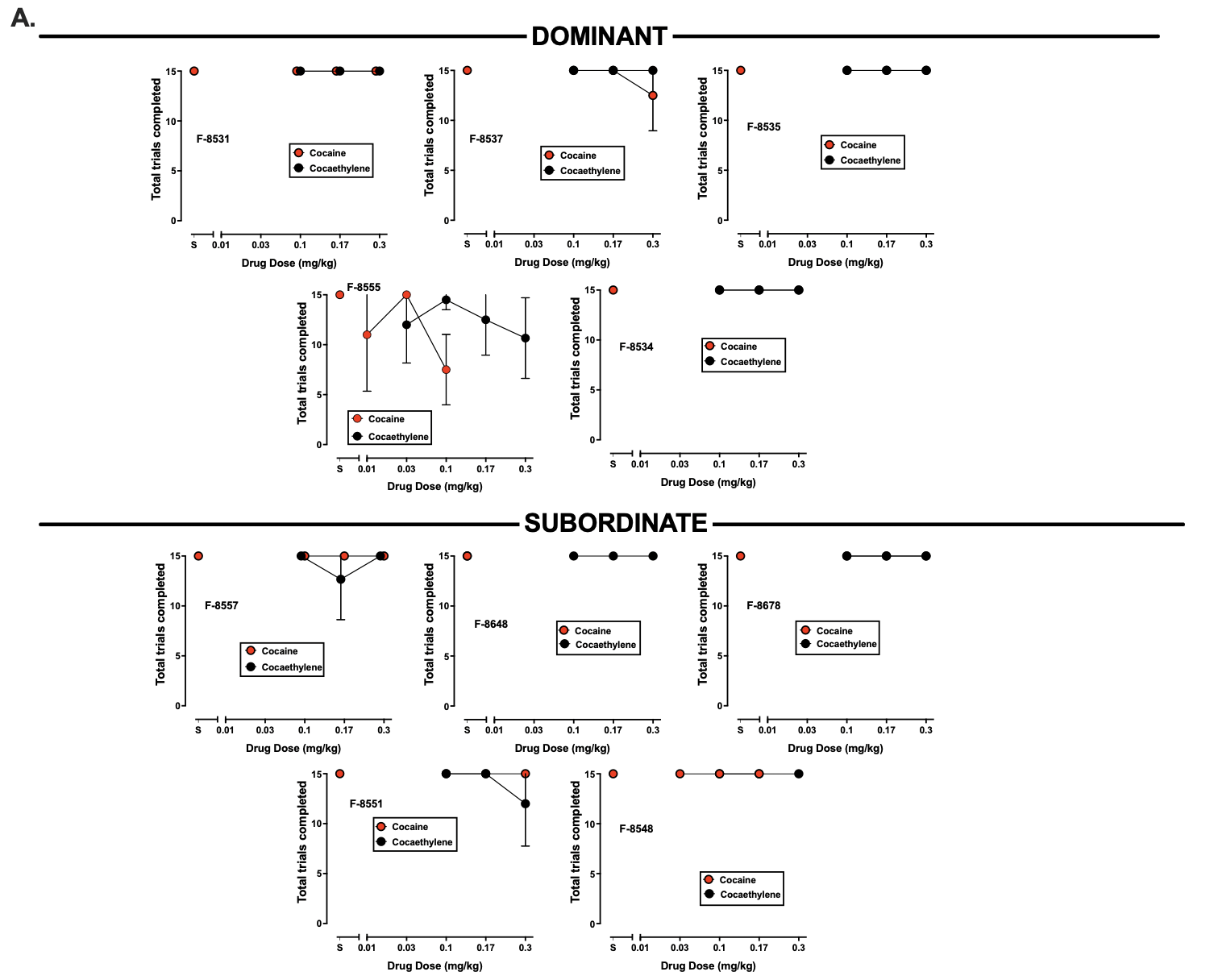


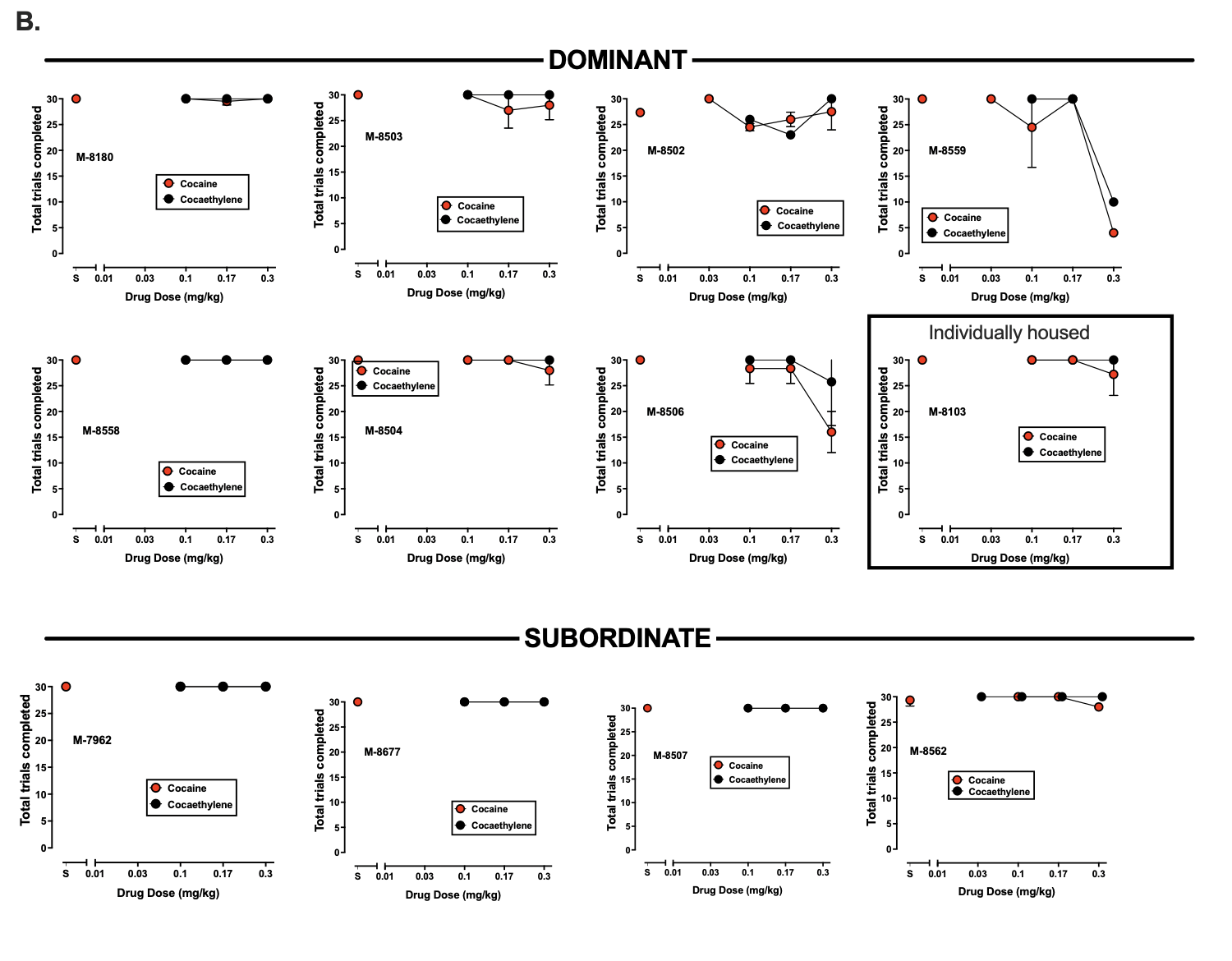


**Supplemental Figure 3:** Percent drug-associated choice as a function of cocaine or cocaine+nicotine dose given as a pretreatment in dominant (top panel) and subordinate (bottom panel) female (**A**) and male (**B**) monkeys. Each point is the mean ± S.D. of at least 2 sessions where a dose of cocaine or cocaine+nicotine was administered prior to saline self-administration sessions. Asterisks depict the dose of cocaine+nicotine at which % drug-associated choice were significantly higher than cocaine alone.


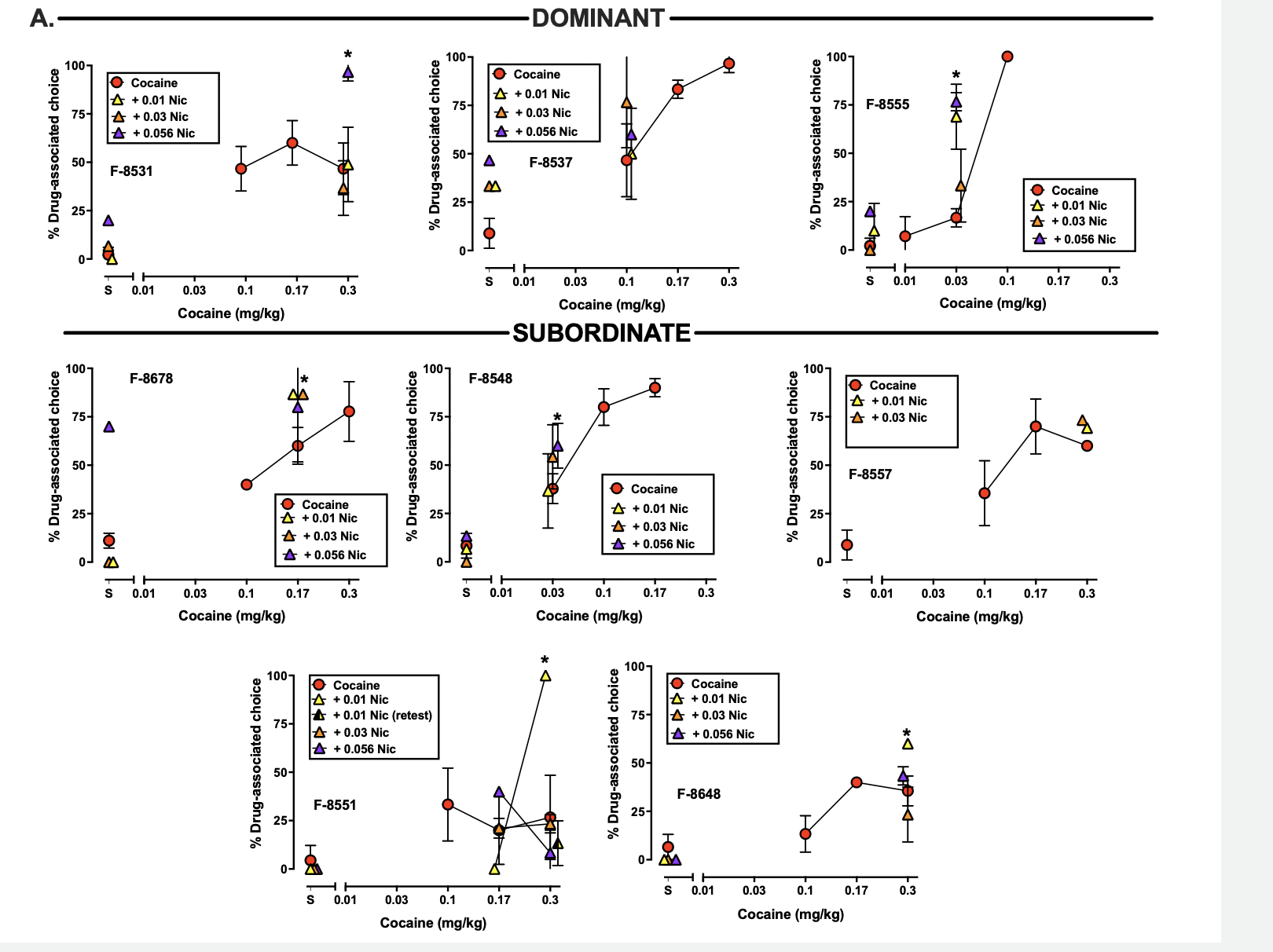


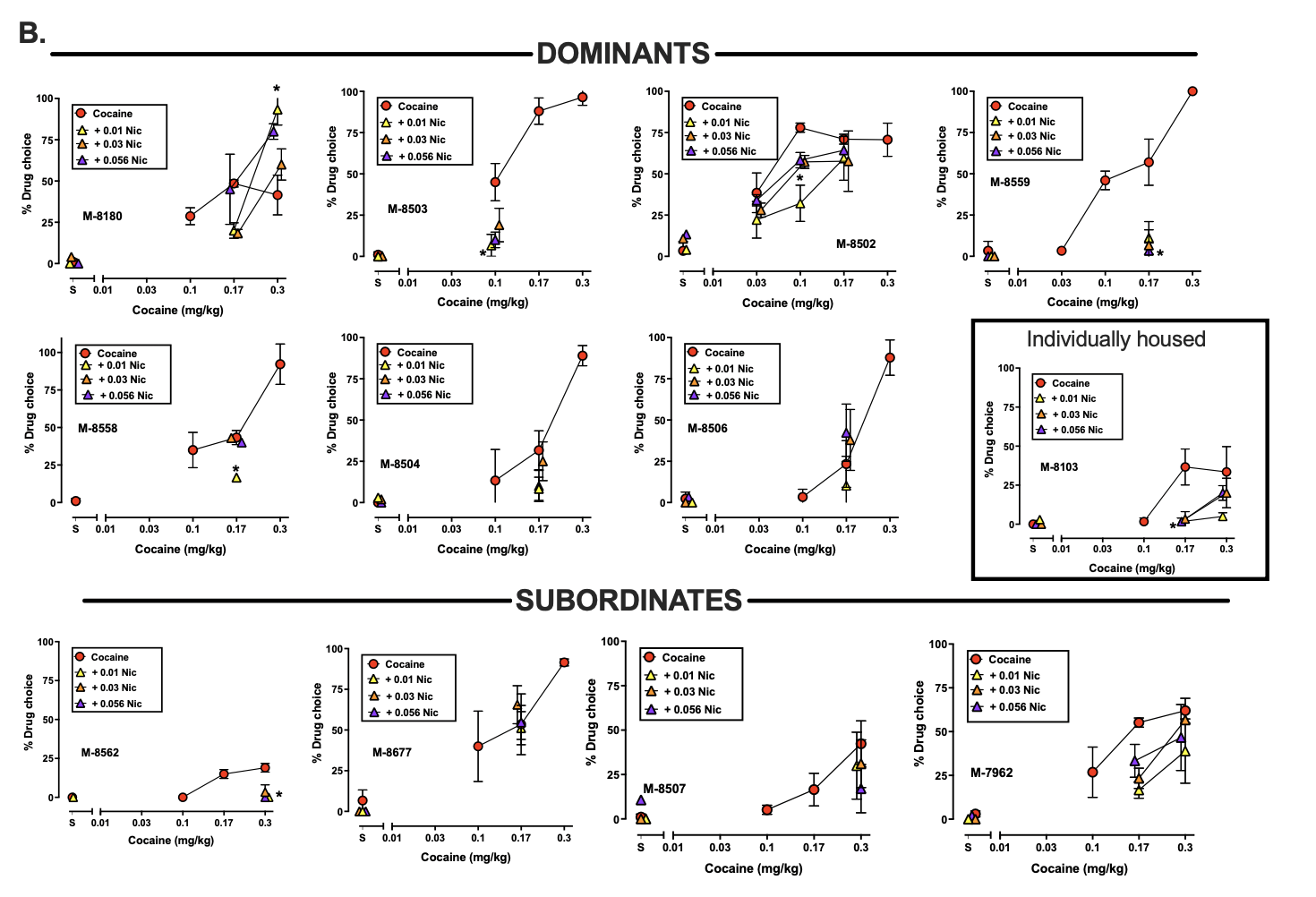


**Supplemental Figure 4:** Percent drug-associated choice as a function of cocaethylene or cocaethylene+nicotine dose given as a pretreatment in dominant (top panel) and subordinate (bottom panel) female (**A**) and male (**B**) monkeys. Each point is the mean ± S.D. of at least 2 sessions where a dose of cocaethylene or cocaethylene+nicotine was administered prior to saline self-administration sessions. Asterisks depict the dose of cocaethylene+nicotine at which % drug-associated choice were significantly higher than cocaethylene alone.


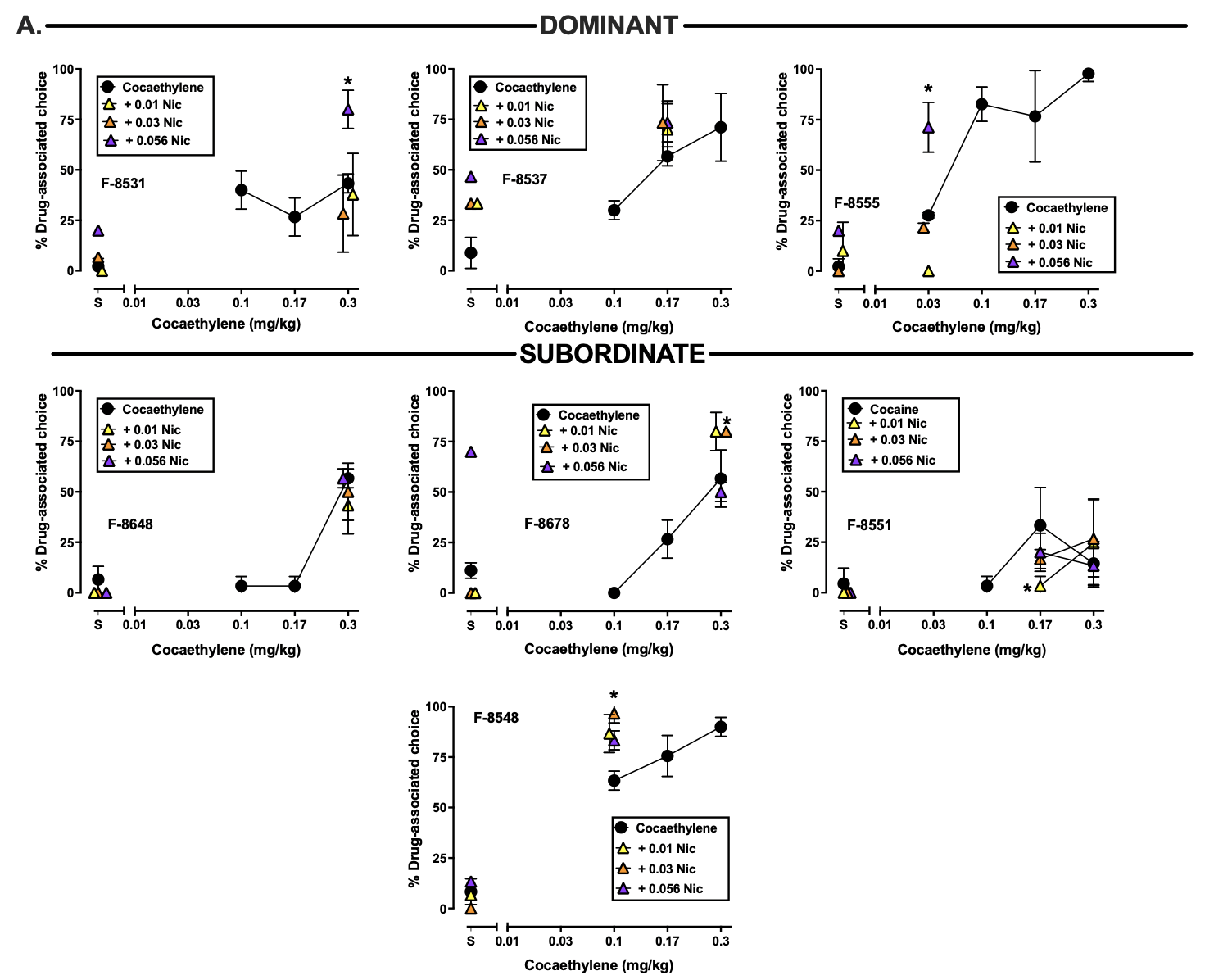


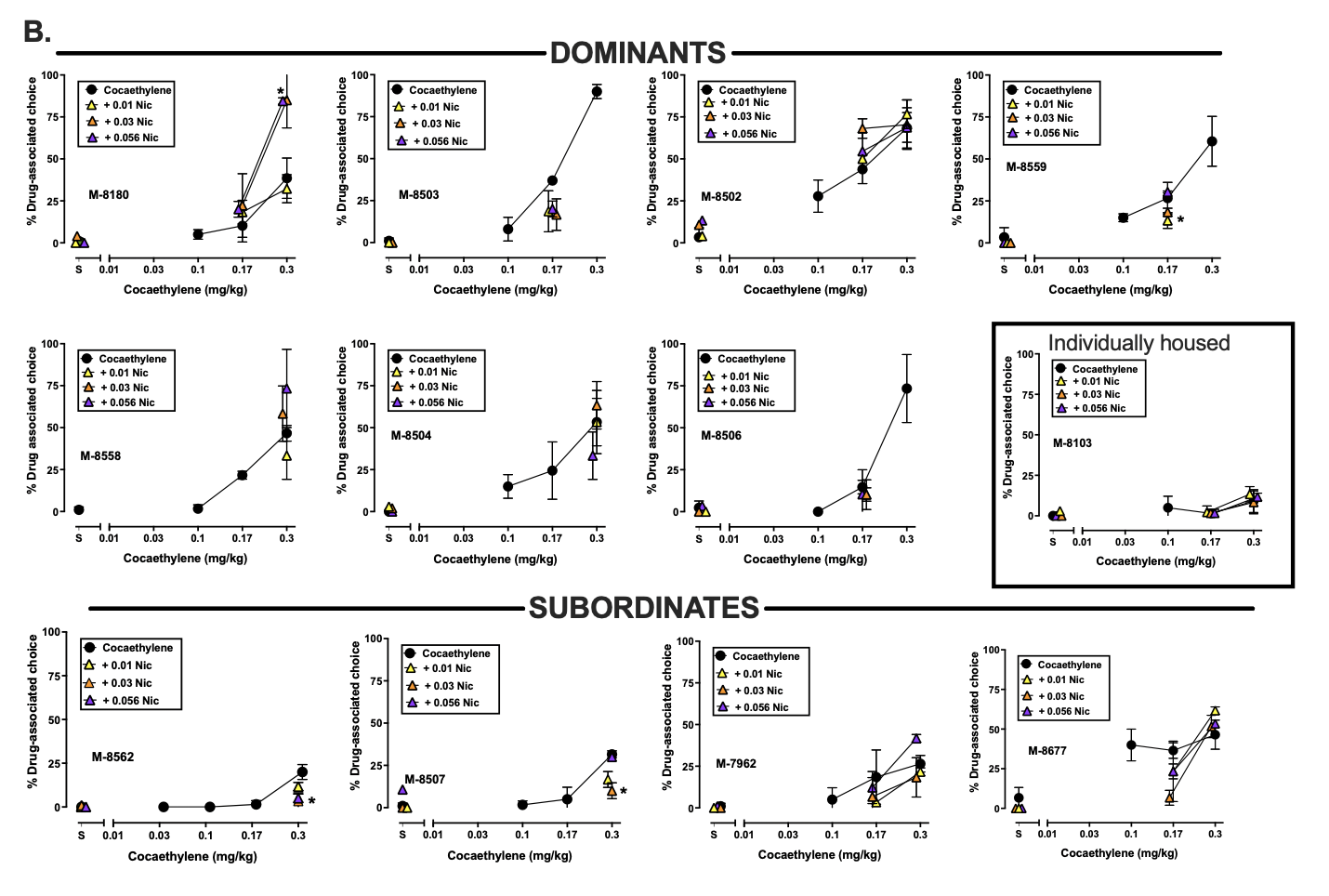


**Supplemental Figure 5:** Number of total trials completed as a function of cocaine or cocaine+nicotine dose given as a pretreatment in dominant (top panel) and subordinate (bottom panel) female (**A**) and male (**B**) monkeys. Each point is the mean ± S.D. of at least 2 sessions where a dose of cocaine or cocaine+nicotine was administered prior to saline self-administration sessions. Note differences in ordinate in males and females.


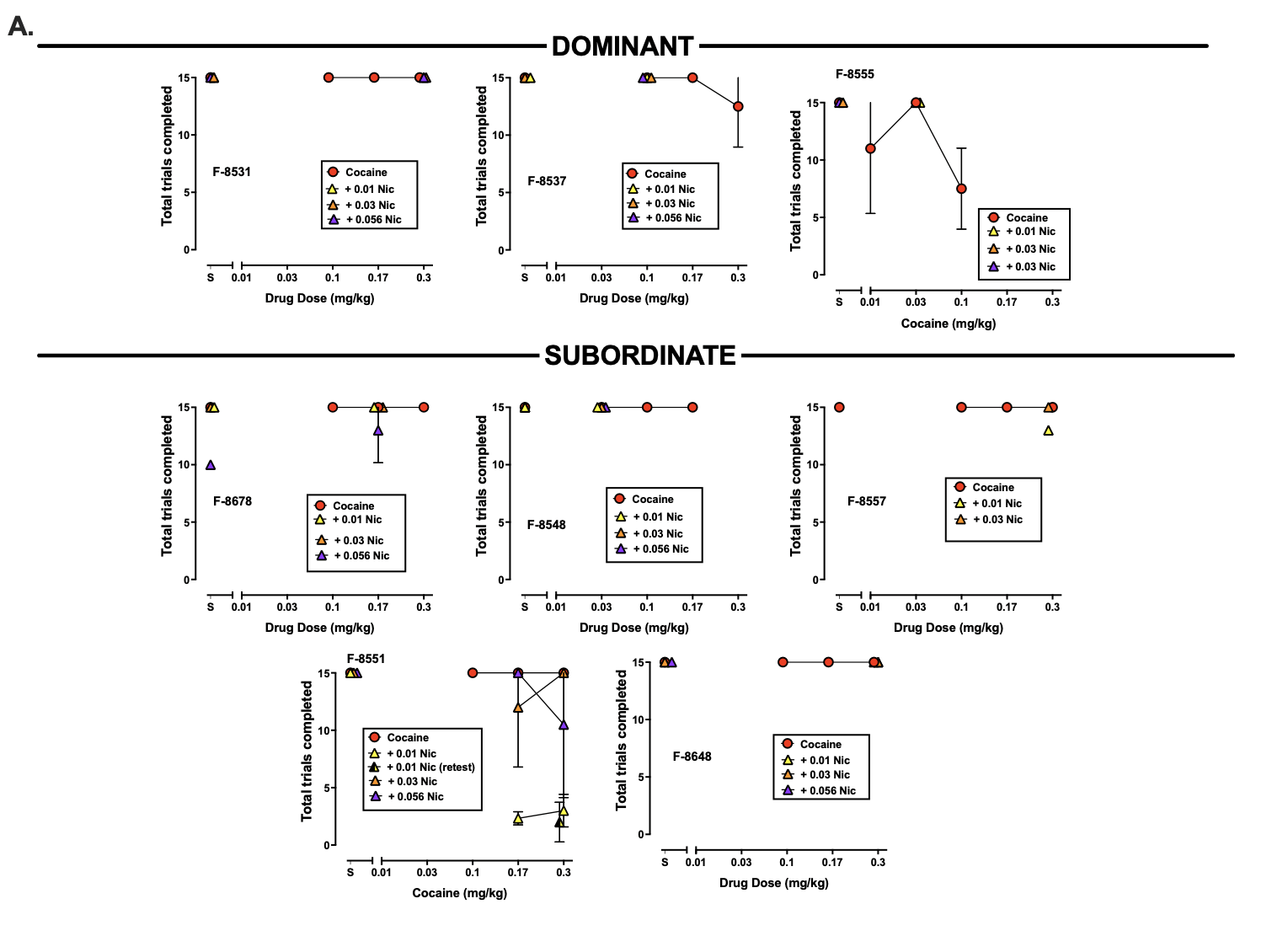


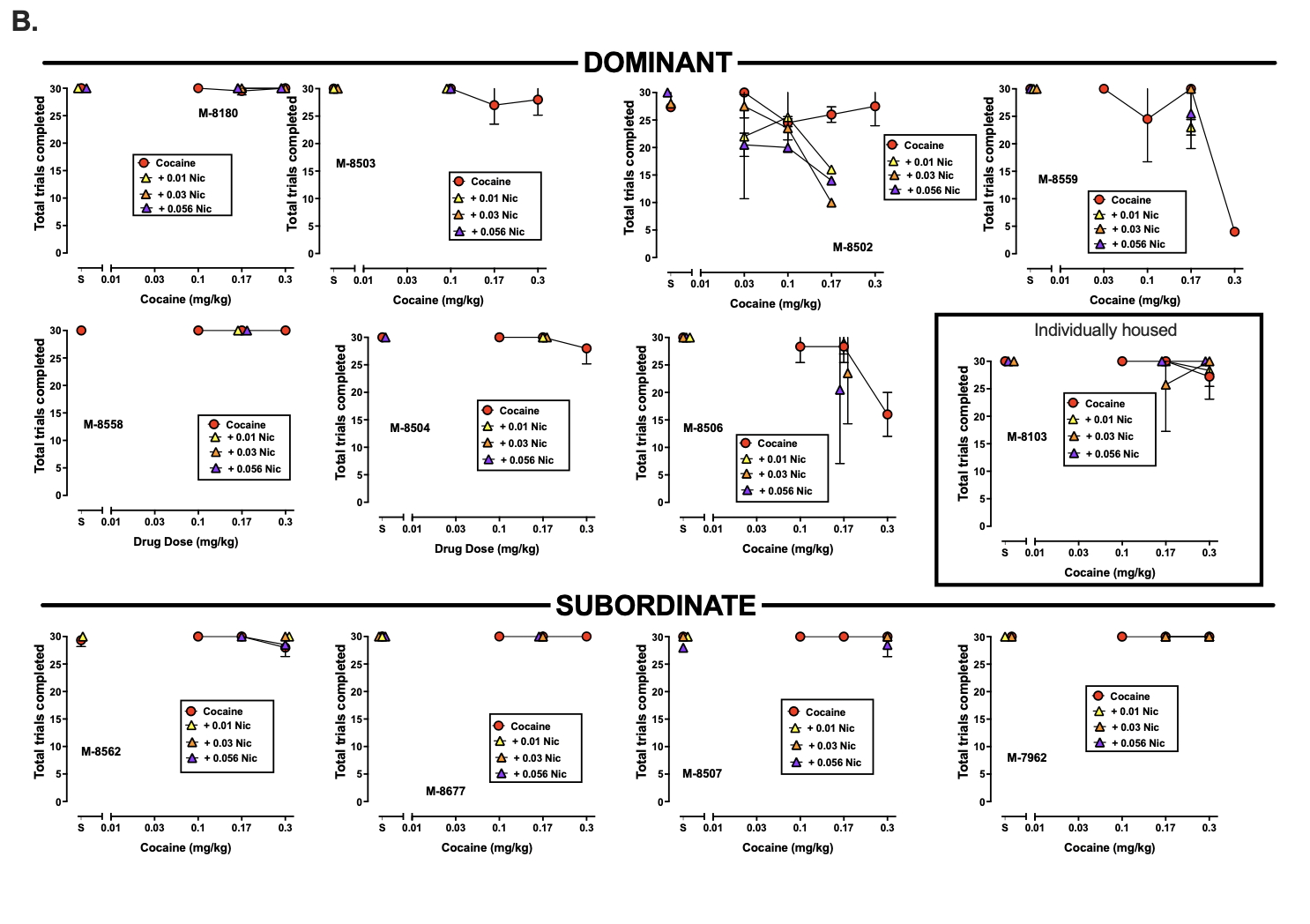


**Supplemental Figure 6:** Number of total trials completed as a function of cocaethylene or cocaethylene+nicotine dose given as a pretreatment in dominant (top panel) and subordinate (bottom panel) female (**A**) and male (**B**) monkeys. Each point is the mean ± S.D. of at least 2 sessions where a dose of cocaethylene or cocaethylene+nicotine was administered prior to saline self-administration sessions. Note differences in ordinate in males and females.


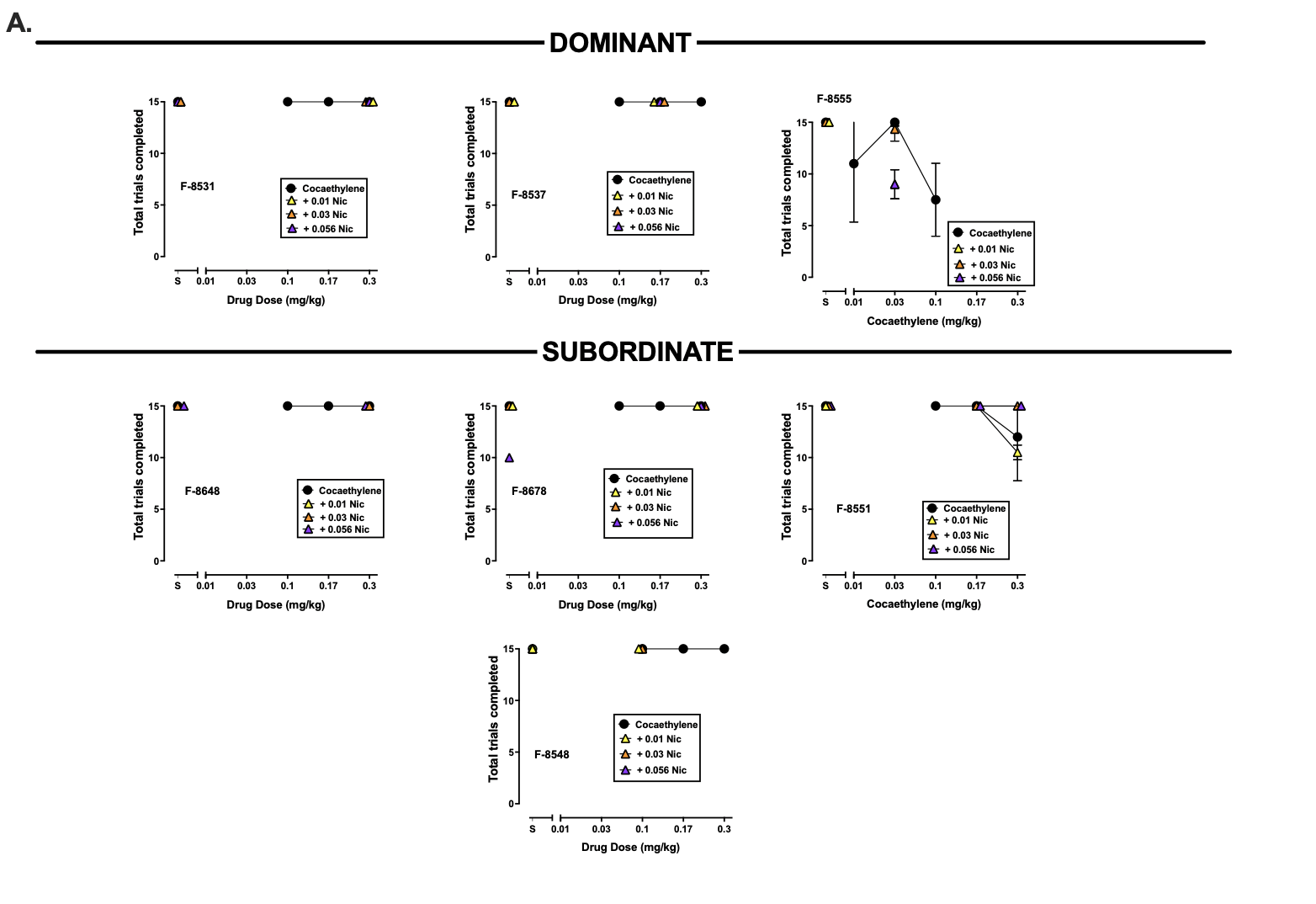


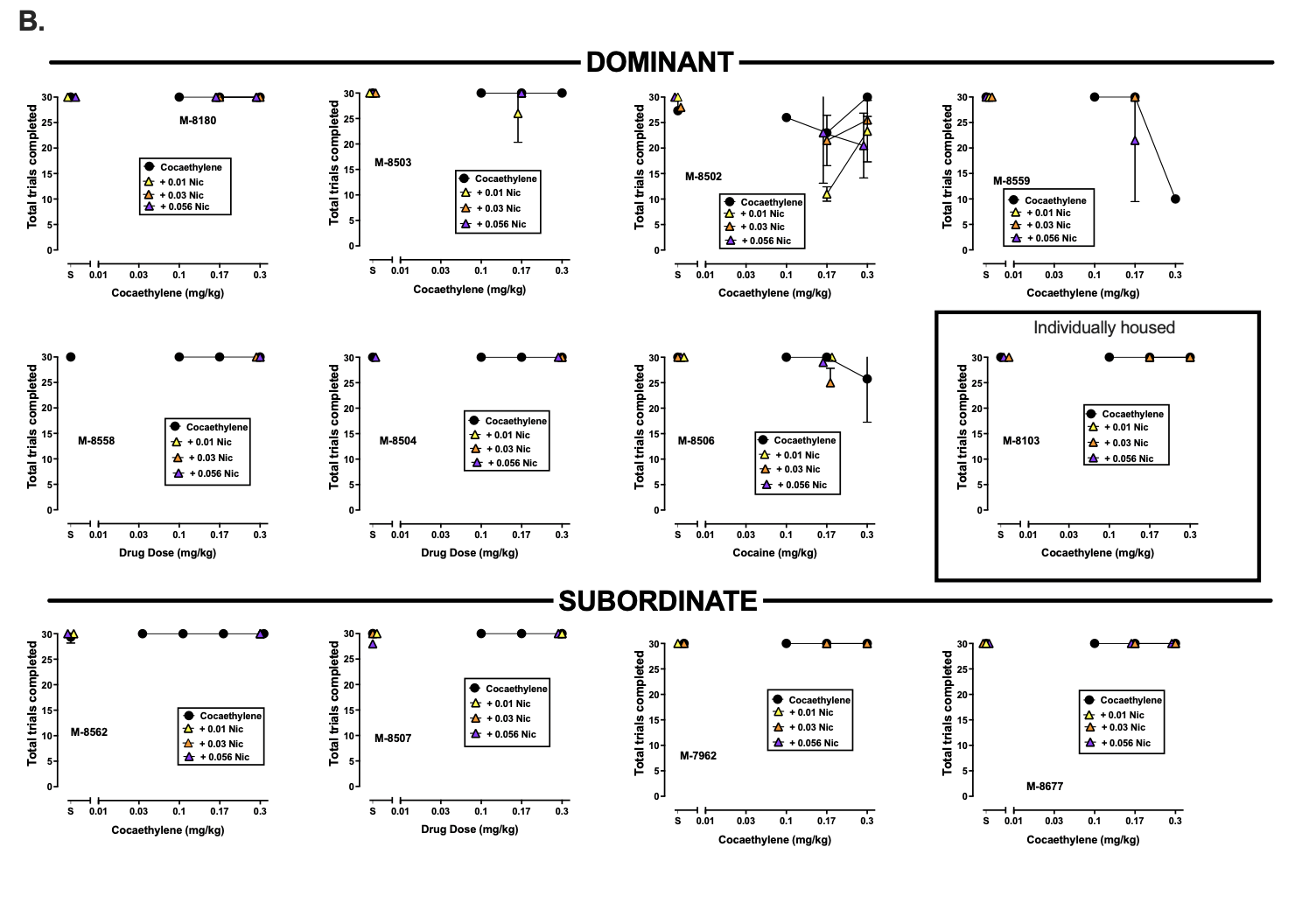

Supplement: Supplementary file 1 [file Data_Sheet_1.docx]
